# An evolutionary perspective on the behavioral consequences of exogenous oxytocin application

**DOI:** 10.3389/fnbeh.2013.00225

**Published:** 2014-01-17

**Authors:** R. Becket Ebitz, Michael L. Platt

**Affiliations:** ^1^Department of Neurobiology, Stanford University School of MedicineStanford, CA, USA; ^2^Department of Neurobiology, Duke University School of MedicineDurham, NC, USA; ^3^Department of Evolutionary Anthropology, Duke UniversityDurham, NC, USA

**Keywords:** oxytocin, social interactions, affiliation, vigilance, decision making, prosociality

## Abstract

Oxytocin (OT) is released in response to social signals, particularly positive ones like eye contact, social touch, sexual behavior, and affiliative vocalizations. Conversely, exogenous delivery of OT has diverse behavioral effects, sometimes promoting affiliative and prosocial behaviors, but sometimes suppressing them. Here, we argue that one unifying interpretation of these diverse effects is to view OT as an evolutionarily conserved physiological signal indicating affiliative interactions and predicting their behavioral consequences. In this model, OT regulates the way information about the social environment accesses the neural circuitry responsible for social behavior, thereby shaping it in sometimes counter intuitive but adaptive ways. Notably, prosociality is not always the most adaptive response to an affiliative signal from another individual. In many circumstances, an asocial or even antisocial response may confer greater fitness benefits. We argue that the behavioral effects of exogenous OT delivery not only parallel the behavioral effects of affiliative interactions, but are themselves adaptive responses to affiliative interactions. In support of this idea, we review recent evidence that OT does not unilaterally enhance social attention, as previously thought, but rather can reduce the typical prioritization of social information at the expense of other information or goals. Such diminished social vigilance may be an adaptive response to affiliative social interactions because it frees attentional resources for the pursuit of other goals. Finally, we predict that OT may mediate other behavioral consequences of social interactions, such as reduced predator vigilance, and argue that this is a rich avenue for future behavioral and neurobiological study.

## Introduction

Oxytocin (OT) is a mammalian neuromodulatory hormone that is released during social behavior. OT and its non-mammalian homologues are critical for parturition and egg laying in mammals and non-mammals alike (Insel and Young, 2001; Donaldson and Young, 2008) and are involved in a wide array of courtship and sexual behaviors in species as diverse as snails (Van Kesteren et al., 1995), stickleback fish (Kleszczynska et al., 2012), and humans (Murphy et al., 1987). In some species, including sheep (Kendrick et al., 1991) and humans (Murphy et al., 1987), sexual stimulation is sufficient to cause the release of OT. These nonapeptides are also endogenously released following child birth in both placental mammals (Donaldson and Young, 2008) and marsupials (Parry et al., 1996).

In mammals, the suite of social behaviors that cause OT release includes many different affiliative social signals. A “social signal” is a feature of or gesture made by a social actor (“signaler”) that conveys information about the state or future behavior of the signaler to another social actor (“receiver”). When the information contained in the signal is veridical, accurately reflecting the state of the signaler, these signals are “honest”. Thus, honest affiliative social signals provide a receiver with information about the likelihood of prosocial (vs. antagonistic) interactions with the signaler (Crockford et al., 2008). The affiliative social signals that result in OT release include touch in the mouse (Stock and Uvnäs-Moberg, 1988; Agren et al., 1995); social proximity, huddling, and grooming in the tamarin (Snowdon et al., 2010); and grooming in the chimpanzee (Crockford et al., 2013). In humans, social touch (Holt-Lunstad et al., 2008) and participating in parental care (Gordon et al., 2010) predict increased peripheral levels of OT, as does receipt of affiliative vocalizations (Seltzer et al., 2010) and interspecies eye contact (Nagasawa et al., 2009). Taken together, current research suggests an evolutionarily conserved link between receipt of affiliative social signals and OT release in mammals. This link has become elaborated during primate evolution, extending from its ancestral role in sexual and maternal behavior to signal a wider array of affiliative social cues such as eye contact and vocal communication.

In stark contrast, the effects of OT on the receiver—its proximate function—are diverse. Exogenous delivery of OT is sufficient to induce maternal behavior in rats (Pedersen and Prange, 1979) and sheep (Kendrick et al., 1991) and pair bonding in monogamous voles (Williams et al., 1994). Exogenous OT delivery also promotes a wide array of social behaviors such as flocking in the zebra finch (Goodson et al., 2009), reward sharing in macaques (Chang et al., 2012) and marmosets (Smith et al., 2010), trusting decisions in humans (Kosfeld et al., 2005), affiliative behaviors such as huddling and grooming in squirrel monkeys and marmosets (Winslow and Insel, 1991; Smith et al., 2010), and eye gaze in humans and macaques (Guastella et al., 2008a; Andari et al., 2010; Gamer et al., 2010; Ebitz et al., 2013).

Together, these findings are consistent with the hypothesis that OT serves largely prosocial functions. However, exogenous OT also promotes social behaviors that are not prosocial or affiliative (Bartz et al., 2011b). For example, OT promotes reward withholding in macaques in some contexts (Chang et al., 2012), reduces trust in some patient populations (Bartz et al., 2011a), increases some negative social emotions in humans (Shamay-Tsoory et al., 2009), reduces the attentional salience of social cues (Ebitz et al., 2013), promotes the punishment of out-group members in humans (De Dreu et al., 2011), and increases aggression in dominant male squirrel monkeys (Winslow and Insel, 1991).

Current unifying theories of OT function in primates have been largely concerned with studies of the behavioral consequences of exogenous OT delivery. In particular, two major models have emerged from these findings: the prosociality hypothesis (Kosfeld et al., 2005; Striepens et al., 2011) and the interactionist component process model (Bartz et al., 2011b). However, these theories explain only the behavioral consequences of exogenous OT delivery, with little consideration of the adaptive function of the peptide. Here, we argue that a unifying theory of OT function in primates should also consider the endogenous causes of OT release and thereby begin to address the fundamental question of what role OT plays in nature. We review the evidence in support of the prosociality hypothesis and the interactionist component process model and evaluate these hypotheses from an evolutionary perspective. This perspective is informed by both the behavioral consequences of OT delivery and the causes of OT release. Throughout, we strive to incorporate relevant observations from the ethological literature. Ultimately, we argue that an evolutionary perspective provides unique new insights into many of the diverse behavioral consequences of exogenous OT delivery.

## The prosociality model of Oxytocin (OT) function

Studies of the behavioral effects of exogenous OT delivery in humans often report that the peptide promotes prosociality (Kosfeld et al., 2005; Guastella et al., 2008b; Striepens et al., 2011). The term “prosociality” refers to behaviors that are beneficial to a social partner. Behaviors that directly benefit the health of social partners such as resource sharing or cooperation towards a common goal are prosocial. Affiliative gestures such as social touch are prosocial because they reduce the stress responses of others (Dunbar, 2010). While many diverse behaviors can be categorized as prosocial, exogenous delivery of OT promotes a surprising diversity of them in humans. OT promotes resource sharing (Kosfeld et al., 2005), eye contact (Guastella et al., 2008a; Andari et al., 2010; Gamer et al., 2010), and positive social signals during conflict (Ditzen et al., 2009). Similarly, OT delivery promotes social contact in marmosets (Smith et al., 2010) and voles (Williams et al., 1994), as well as resource sharing (Chang et al., 2012) and eye contact (Ebitz et al., 2013) in rhesus macaques. This is not an artifact of exogenous manipulation, but rather may reflect the endogenous function of the peptide. For example, variation in the OT receptor gene also predicts resource-sharing decisions (Israel et al., 2009). Thus, OT has multiple, evolutionarily conserved prosocial effects, suggesting that one essential function of the peptide is the promotion of prosociality.

The hypothesis that OT evolved to promote prosociality may have its roots in early psychological theories of prosociality. In one of the first textbooks of social psychology, Mcdougall (1908) hypothesized that prosociality was a natural consequence of parental instincts, the result of “tender emotions” originally directed towards offspring that were later co-opted to promote the helping of others. OT would seem a likely substrate for these tender emotions. OT is critically involved in mammalian maternal behavior (Pedersen and Prange, 1979; Kendrick et al., 1987) and, in humans, endogenous OT levels are correlated with individual differences in infant-directed care behaviors in both men and women (Gordon et al., 2010). However, as is true of most correlative studies of endogenous OT levels, it is unclear whether OT is the cause or consequence of these affiliative parental behaviors.

While many aspects of maternal behavior are prosocial or affiliative, maternal behaviors are not uniformly prosocial. Rather, maternal behavior can also include non-social behaviors such as nest building (Pedersen and Prange, 1979) and antisocial behaviors such as increased aggression and territoriality toward other adults (Maestripieri, 1992). These postpartum changes in behavior have adaptive functions. For example, increased aggression and territoriality protects the vulnerable infant from predation and aggression by conspecifics (Maestripieri, 1992). Selection pressures thus do not strictly favor tender postpartum changes in behavior, but rather behaviors that enhance offspring survival. Moreover, just like prosocial maternal behaviors, nest building (Pedersen and Prange, 1979) and maternal aggression (Ferris et al., 1992) can be induced by central delivery of OT.

**Figure 1:**
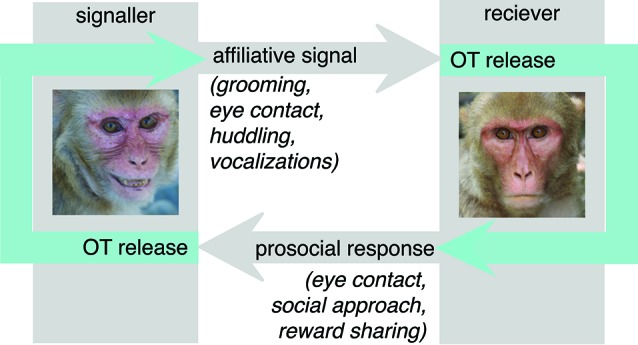
**In nature, OT is released in the context of dyadic interactions.** In particular, receipt of affiliative social signals such as eye contact, grooming, social proximity and affiliative vocalizations provokes OT release. The prosociality hypothesis argues that endogenous OT largely functions to release these same behaviors in the receiver, increasing eye contact, proximity seeking and social approach behaviors directed back towards the signaler. These actions would then provoke OT release from the signaler, leading to a cascade of reciprocal affiliation and prosociality. While this is likely to be an important mechanism in the formation of social bonds, an inflexibly reciprocating response may not be adaptive in all circumstances.

One theoretical concern with the prosociality model is the observation that it is reciprocal when the endogenous causes for OT release are considered (Figure 1). Because OT is released in response to affiliative cues from a signaler, it is unlikely to invariably promote prosocial behavior in the receiver. While prosociality is frequently a reasonable response to the receipt of affiliative social signals, there are circumstances in which prosociality is not the most adaptive response. Prosociality can be costly, either in terms of time spent, energetic expenditure, or resources lost to sharing. Thus, natural selection would not favor indiscriminate prosociality, but rather promote the judicious deployment of prosocial behavior, appropriate to the individual and social context.

Consider the case of an encounter with an individual from outside one’s group. Gregarious animals divide their social worlds into in-group members (social partners who belong to the same group) and out-group members (individuals who belong to other groups). Relationships with in- and out-group members are different in several ways. In-group members have many opportunities to interact, and thus to reciprocate prosocial behavior. For example, sharing resources with in-group member today may result in grooming tomorrow. Conversely, out-group members are much less likely to interact in the future, and thus have fewer opportunities to reciprocate today’s prosocial gestures.

In addition to more frequent opportunities for reciprocation, in-group members also have comparatively greater access to information about each other’s propensity towards prosociality. Having knowledge of other individuals’ past interactions with others is sufficient to promote prosociality and cooperation in a population (Nowak and Sigmund, 1998, 2005). In-group members, due to their frequent proximity, have many opportunities to observe each other interact and gather information about each other, while little is likely to be known about an out-group member. Indeed, prosocial behaviors are more commonly directed towards in-group members than out-group members (Hewstone et al., 2002). Moreover, the effects of OT on prosocial decisions are mediated by in/out group status (De Dreu et al., 2011).

Even beyond this specific circumstance, too much prosociality can hinder rather than help fitness. In evolutionary game theory, simulations are used to model repeated interactions between dyads within a population, in which the consequences for fitness of each interaction are derived from game theoretic payoff matrices (Axelrod and Hamilton, 1981; Nowak and Sigmund, 2005). The fittest individuals survive to pass their strategies on to the next generation. These simulations model behavior at a macro level: they address the outcome of interactions, rather than the process of interactions, and look at the expression of various strategies in the population as it evolves over time. Thus, these studies provide insight into the social strategies that are most likely to be favored by natural selection, given the parameters of the simulations and payoff matrices. In population-level simulations of the sort of economic games in which OT can increase cooperation (Declerck et al., 2010) and trust (Kosfeld et al., 2005), the most adaptive strategies are rarely the most prosocial (Axelrod and Dion, 1988; Dacey and Pendegraft, 1988; Nowak and Sigmund, 1998, 2005). In many cases, evolution may favor deception and defection. It is important to note that in the natural world that these games simulate, each of these decisions would be predicated on the same conspecific behaviors, such as approach, eye contact, and other affiliative signals. The hardwired release of prosocial responses would result in an “always cooperate” strategy.

In such games, however, the strategies that thrive are rarely strictly prosocial. Rather, winning strategies, though strongly dependent on simulation parameters, can involve defection in response to a partner’s previous defection (Axelrod and Hamilton, 1981), permanent defection following a single antisocial act from a partner (Dacey and Pendegraft, 1988), defection in response to changes in one’s own fitness level (Axelrod and Dion, 1988), or defection dependent on the cost-to-benefit ratio of the reciprocal act (Nowak and Sigmund, 1998). A hardwired mechanism promoting prosociality in response to affiliative signals would be selected against, unlikely to outcompete other strategies for expression, even when the majority of individuals within that population were initially prosocial since it takes only a few defectors to outcompete a population of strict cooperators (Axelrod and Hamilton, 1981).

While these are largely theoretical concerns, the evidence against a solely prosocial function for OT does not end here. Rather, exogenous delivery of OT does not uniformly increase prosociality. In humans, OT reduces prosocial decisions towards out-group members (De Dreu et al., 2011) and reduces prosocial decisions in persons with borderline personality disorder (Bartz et al., 2011a). In non-human primates, OT reduces reward-sharing choices in some decision contexts, such as when monkeys have to choose between delivering reward to themselves or giving reward to another monkey (Chang et al., 2012). Similarly, in freely behaving squirrel monkeys, OT increases both affiliation and aggression (Winslow and Insel, 1991), depending on the dominance status of the monkey treated with OT. Thus, OT does not consistently promote prosociality, but rather can have complex and seemingly contradictory effects on social behavior.

Despite these challenges, insofar as reciprocal prosociality is expressed in nature it seems probable that OT is involved. Reciprocal prosociality is the very definition of social bonds, which have profound consequences for health and mating opportunities. Thus, disruption of the endogenous OT system may have profound consequences for both social relationships and health. Indeed, blockade of the OT system blunts the species-typical appearance of social bonds in marmosets (Smith et al., 2010) and voles (Williams et al., 1994). Moreover, knockout mice lacking the OT receptor gene have severely reduced social bonds, engaging in fewer proximity soliciting behaviors and failing to develop familiarity with other mice (Winslow and Insel, 2002; but see Crawley et al., 2007). Autism, a developmental disorder characterized by reduced social engagement, has been associated with changes in the OT receptor gene (Wu et al., 2005; Jacob et al., 2007), depressed OT levels in plasma (Modahl et al., 1998), and with alterations of the peptide’s structure (Green et al., 2001). While there is correlational evidence for a link between the endogenous OT system and the ability to establish and maintain social bonds, additional work is needed to determine whether OT is truly necessary and sufficient for reciprocal prosociality. In particular, the development of an animal model of reciprocal prosociality would allow for the blockade and rescue of OT function required to address this question.

## The interactionist component process model of Oxytocin (OT) function

The interactionist component process model was developed to address the diverse and complex effects of exogenous OT delivery (Bartz et al., 2011b). According to this hypothesis, OT has different effects, depending on context and individual, due to neuromodulatory effects on the component computations that shape these decisions. Complex social behaviors are unlikely to depend on a single neural substrate. Rather, they require the involvement of many cortical and subcortical structures to track social percepts, perform theory of mind computations, evaluate rewards, select actions, and even regulate arousal. Complex, context-dependent effects of OT delivery could arise simply from unidirectional effects on these component processes. The original formulation of this model posits several specific component processes; specifically that OT enhances social salience, promotes affiliative motivations, and reduces social anxiety. Broadly, this model provides a coherent framework that accounts for the diverse behavioral observations that prove challenging for the prosociality model.

This model thus provides a mechanistic account by which OT may generate disparate behaviors but does not address the ultimate function of OT. In particular, the component process model does not explain why OT affects these particular component processes in different ways. Moreover, because there is a paucity of data on the distribution and function of OT receptors within the primate brain, the model’s component processes are based on the categorization of the existing literature into conceptual groups, rather than on a neurobiological framework. Additional work on the distribution of OT receptors in humans and other primates is needed to inform future modifications to this model.

Here, we argue that one particular component process included in the original model should be updated, drawing on recent behavioral evidence, knowledge of the distribution and function of endogenous OT activity, and our hypothesis that the behavioral effects of OT delivery are adaptive responses to affiliative social signals. In particular, we hypothesize that OT should reduce, rather than enhance, the typical salience of social stimuli, in direct contrast to the predictions of the interactionist component process model.

“Salience” is the degree to which a stimulus is likely to be selected by attention: prioritized for processing at the expense of other possible attentional targets. Cues that predict important events in the environment are salient because they attract attention, regardless of current goals. Such cues are behaviorally relevant, either in a deep biological sense or because they have previously proved predictive of biologically relevant events. It should come as no surprise then that social animals are attentionally salient for each other. For example, faces are uniquely salient attentional targets in both humans (Cerf et al., 2009) and rhesus macaques (Ebitz et al., 2013). The critical and unique “social salience” of a face allows for the rapid and error-free detection of conspecifics and likely plays an important role in more complex social behaviors. Monitoring others is a fundamental component of normal social behavior because it allows animals to avoid agonistic interactions and pursue affiliative ones. For primates, the face in particular provides critical information about the identity and behavioral state of social partners that can be used to guide behavior (Leopold and Rhodes, 2010).

**Figure 2:**
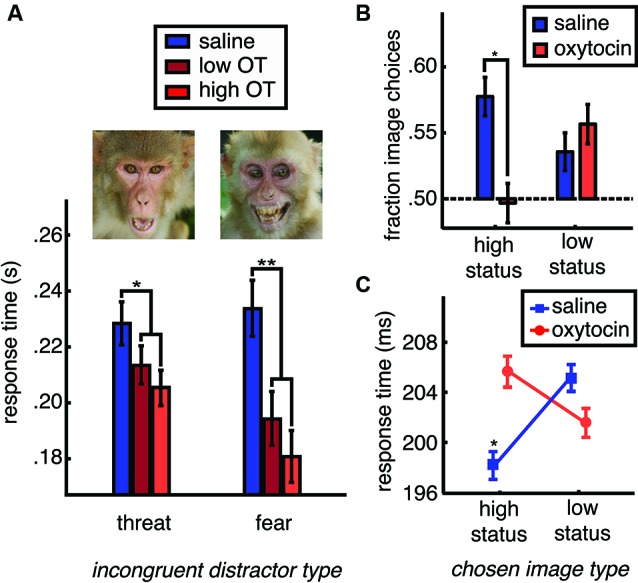
**OT reduces species typical social vigilance in the rhesus macaque.** Rhesus macaques are typically vigilant for specific classes of others: those with high status and those displaying emotional expressions such as fear or threat. **(A)** One way to measure social vigilance is to examine how much the appearance of task-irrelevant social information interferes with task performance. When fearful and threatening faces are presented in competition with rewarded targets, they slow response times, increasing the latency of eye movements towards rewarded targets (blue bars). However, OT dose dependently reduces the typical slowing of task response times caused by task-irrelevant emotionally expressive faces (shades of red). OT thus reduces the distraction typically caused by emotionally expressive faces. **(B)** Another way to examine social vigilance is to look at choices to gather social information. Typically, rhesus macaques will choose to view high status others. However, OT treatment reduces choices to view high status faces. **(C)** High status face choices are typically accompanied by fast response times (* indicates faster response times for this category compared to low status faces (plotted), female perinea (not plotted), and gray square images (not plotted)). However, after OT treatment, choices to view high status faces are no faster than choices to view low status faces or the other image categories. Figures adapted from Ebitz et al. (2013).

While OT promotes gaze to the eye region of the face (Guastella et al., 2008a; Andari et al., 2010; Gamer et al., 2010; Ebitz et al., 2013), and social gaze to both photographs (Ebitz et al., 2013; Parr et al., 2013) and live conspecifics (Chang et al., 2012), OT conversely obliterates the attentional salience of face images (Ebitz et al., 2013). In a recently reported study, face and non-face distractor images were briefly flashed in the periphery while monkeys made eye movements to targets in order to receive fluid reward. Spatially incongruent distractor stimuli interfered with saccades to rewarded targets, causing slower response times compared to when no distractors were present. Moreover, saccades to rewarded targets were even slower and less accurate following face distractors, compared to non-face distractors. This response time slowing effect was the strongest for images of emotional faces. OT, however, flattened the typical attentional salience of faces in general, causing a dose-dependent reduction in response time slowing following face distractors. This effect was particularly strong for emotional face distractors (Figure 2A). Even at longer presentation times, OT blunts species-typical gaze to emotional faces in the rhesus macaque (Parr et al., 2013) and human (Domes et al., 2012). Moreover, like many other animals that live in hierarchical societies (Mcnelis and Boatright-Horowitz, 1998), rhesus macaques make sacrifices to monitor high status others (Deaner et al., 2005), but OT blunts this prioritization of information about dominant monkeys (Figures 2B, C).

Thus, OT seems to reduce, rather than enhance, the attentional salience of critical social signals. From an adaptive perspective, this makes sense. Social attention exacts time and opportunity costs so it is maladaptive to maintain a state of high social vigilance when the absence of social threat has been communicated through affiliative signals. Adjusting the attentional priority of social information in response to these signals would conserve energetic and attentional resources for the pursuit of other goals, such as foraging. However, the studies reviewed above do not provide an exhaustive assessment of the effects of OT on the salience of social stimuli. It remains possible that OT may enhance the attentional salience of some social cues in some circumstances. OT may enhance the attentional salience of happy facial expressions, for example, or selectively enhance the attentional salience of threatening facial expressions for new mothers.

At first glance, it seems difficult to place these finding in the context of previous reports that OT promotes gaze towards the eye region of faces (Guastella et al., 2008a; Andari et al., 2010; Gamer et al., 2010; Ebitz et al., 2013) and towards live conspecifics (Chang et al., 2012). However, it is important to draw a distinction between the mechanisms that rapidly direct attention towards social stimuli (social salience) and those that maintain attention towards a social stimulus once it has been initially detected (social gaze). Like other forms of attention, social attention need not be a unified process, but rather may rely on multiple competencies that together function to direct processing resources towards social stimuli. It is possible that OT has opposing effects on the mechanisms that direct and sustain social attention. Unfortunately, none of studies indicating that OT promotes social gaze have included the necessary nonsocial controls to show that OT has a specifically social effect on gaze, rather than a more general, low-level effect, such as promoting gaze to high contrast image features. Moreover, in our own work, we found that OT promoted gaze to both face and non-face images, but had more specific effects on the attentional salience of social images (Ebitz et al., 2013).

Many alternative explanations of the effect of OT on social gaze have been posed. In particular, OT may make gaze less goal directed, thereby enhancing feature-dependence (Schulze et al., 2011), it may reduce the anxiety that typically inhibits eye gaze (Averbeck, 2010), or it may reduce the efficacy of gaze in collecting social information, in which case longer viewing durations may be a compensatory strategy (Ebitz et al., 2013). Regardless of the mechanism, OT seems to fundamentally shift the purpose of social gaze. Rather than being rapidly deployed to salient emotions and identities after OT delivery, social gaze is sustained and directed towards the eyes of others.

The suppression, rather than enhancement, of social salience following OT release is consistent with known neural effects of OT, particularly on the amygdala. The amygdala serves an important function in vigilance. Simply stimulating the amygdala of the anesthetized cat results in a searching response that resembles attentive scanning (Ursin and Kaada, 1960). Amygdala signaling modulates activity in extrastriate cortex in a way that resembles classic top-down attentional signals in primates (Morris et al., 1998) and the amygdala is necessary for the enhanced detection of emotional stimuli relative to non-emotional stimuli (Anderson and Phelps, 2001). On a trial-by-trial basis, activity of single neurons in the amygdala predicts attentional deployment to rewarded stimuli (Peck et al., 2013). Moreover, many amygdala nuclei contain single neurons that respond to faces, including the central (output) nucleus, as well as the more associative basolateral and medial amygdala (Brothers et al., 1990; Gothard et al., 2007; Hoffman et al., 2007). These neurons are tuned to particular identities and expressions, such as fearful and threatening faces. The amygdala thus carries signals that parallel the behavioral expression of facial salience: faces elicit greater activity than non-faces, emotionally expressive faces elicit even greater activity, and amygdala activity is associated with and necessary for stimulus prioritization.

OT receptors are particularly prevalent in the primate amygdala compared to many other brain regions (Boccia et al., 2007). Moreover, exogenous OT reduces amygdala responses to salient stimuli in humans (Kirsch et al., 2005; Petrovic et al., 2008; Gamer et al., 2010) and both amgydala and behavioral responses in mice (Viviani et al., 2011; Knobloch et al., 2012). While OT has been reported to blunt activity in one amygdala subregion in an fMRI study (Gamer et al., 2010), it is important to note that the amygdala is not a unitary structure, but rather a collection of interconnected nuclei that make very different contributions to behavior (Ursin and Kaada, 1960). Moreover, many of its intrinsic connections are inhibitory and techniques like fMRI or single unit recordings cannot distinguish between changes in the firing of inhibitory or excitatory neurons. Detailed, local perturbations of OT levels in the mouse report that OT activates inhibitory interneurons, which in turn suppress activity in the central, output nucleus of the amygdala (Huber et al., 2005), but much additional research utilizing local perturbations of OT signaling in the amygdala are needed to fully understand the impact of OT on neuronal activity and associated behavior.

## The effects of exogenous Oxytocin (OT) on social decision-making in primates

The complex and apparently contradictory behavioral effects of exogenous OT may have some unified relationship. It is possible, for example, that OT may simply increase the expression of individuals’ preferred or default strategies in social decision-making (Shamay-Tsoory et al., 2009; Declerck et al., 2013). OT would then increase prosocial behaviors when they are already likely and suppress them further when they are unlikely. This magnification of pre-existing decision preferences could be simply enacted through unified effects on component processes known to be affected by OT (Bartz et al., 2011b). The attentional salience of emotional social cues may be one such component process effect (Ebitz et al., 2013; Parr et al., 2013). Reducing the salience of important social information would make decisions less responsive to the vagaries of the external environment and more dependent on the pre-existing biases of the decision maker.

Decisions in nonsocial domains are made on the basis of the acquisition of sensory information and the acquisition of information about value (Roitman and Shadlen, 2002; Krajbich et al., 2010). Similarly, the acquisition of social information shapes social behavior and decisions (Van Kleef et al., 2010). In other decision-making domains, altering perceptual inputs to decision circuitry provokes specific changes in decisions. For example, altering the quality of perceptual information shapes the rate at which decision processes rise to the threshold for making a decision (Roitman and Shadlen, 2002). The rate of evidence accumulation can be detected in response time: longer latency decisions are associated with slower accumulation processes. Moreover, at least in the domain of value-based decision-making, the accumulation of sensory evidence in favor of a particular decision is gated by attention (Krajbich et al., 2010).

Given these known effects of attention on decision-making in other domains, it is probable that OT-mediated alterations in social salience would have two specific consequences for social decisions. First, if less social information is acquired because OT suppresses social attention, less information about the external social world would be available at the level of the decision circuitry, rendering social decisions less responsive to the external social environment. This could have consequences for social decisions, rendering them either more prone to noise or more consistent with other factors that influence decision circuitry, such as pre-existing biases. Second, a change in social information availability would also be apparent in response time, which would be slower when less social information was accumulated, barring any changes in response threshold (Roitman and Shadlen, 2002).

**Figure 3:**
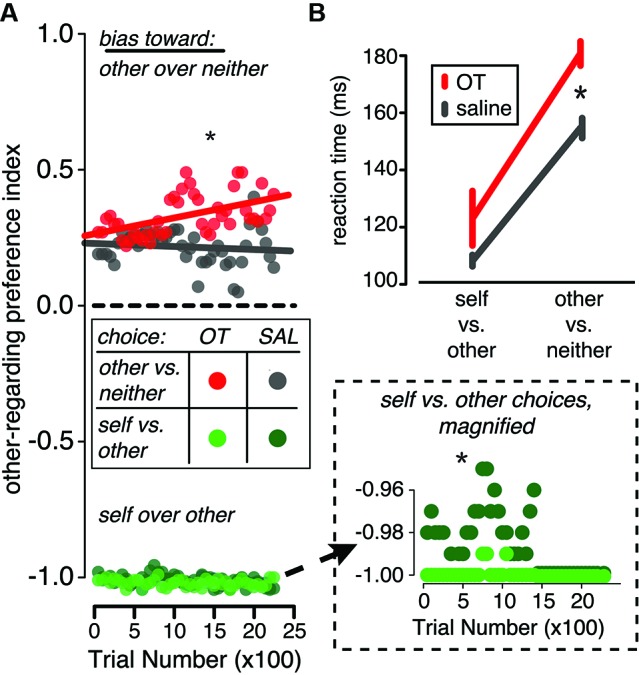
**In a reward-sharing social decision task in the rhesus macaque, OT promotes both pro- and anti-social decisions and increases decision time.** In this task, monkeys were required to choose between donating or withholding rewards given various pairs of choice options (decision contexts). Two decision contexts are depicted here: choices made between rewarding another monkey and rewarding no one (other vs. neither context) and choices made between rewarding self and rewarding the other monkey (self vs. other context). **(A)** In the other vs. neither choice context, OT delivery (red) increased the frequency of prosocial decisions compared to saline delivery (gray). Conversely, when the monkeys chose whether or not to deliver reward to another monkey in the self vs. both context, OT (light green) increased the frequency of selfish choices compared to saline (dark green; *y*-axis magnified in inset). **(B)** OT (red) also slowed response times in both decision contexts compared to saline (gray), but particularly in the other vs. neither context. Figures adapted from Chang et al. (2012).

Across behavioral studies in multiple species, this hypothesis is consistent with the effects of exogenous OT on decision-making. In the rhesus macaque, for example, OT delivery magnifies preexisting biases in social decision making by increasing the frequency of reward sharing when monkeys are already prone to reward sharing and suppressing it when they already prefer not to share rewards (Figure 3A). These changes in decision bias are accompanied by slower reaction times (Figure 3B). Similarly, in the squirrel monkey, OT increases aggressive and sexual behaviors selectively in dominant monkeys, who were already more prone to these behaviors than their subordinate counterparts (Winslow and Insel, 1991). OT may also have similar consequences for social-perceptual judgments. In humans, OT induces positive biases in emotion classification (Di Simplicio et al., 2009) and increases the false alarm rate for identifying faces as familiar (Savaskan et al., 2008; Rimmele et al., 2009).

These observations raise a significant methodological concern for future studies of exogenous OT delivery. Namely, it is possible that apparently unidirectional effects of OT on behavior are not due to a fundamental function of the peptide, but rather to biases in the behavioral context or subjects, which are simply magnified by OT delivery. Thus, future research on the effects of OT on social decisions should employ within-subject manipulations and construct payoff matrices to ensure indifference between decisions at baseline.

Though the idea that OT magnifies pre-existing biases finds support in the existing literature, it is, like the component process model, limited in scope. It addresses only the consequences of exogenous delivery of OT and not the adaptive function of the peptide. Nevertheless, these observations are consistent with our hypothesis that OT promotes adaptive responses to affiliative social signals. Following affiliative social signals, it makes sense for decisions to be less dependent on others’ emotions and more consistent with the actor’s own preferences. When affiliative signals are “honest” (conveying veridical information about the signaler) they indicate that the probability of an agonistic interaction with the signaler is small (Crockford et al., 2008). While social behaviors and decisions are typically highly responsive to others (Van Kleef et al., 2010), social responsivity can be in conflict with an individual’s preferences for particular social decisions or behaviors. However, when the likelihood of a negative or antagonistic response is low, an individual’s preferred social behaviors can be deployed with relative impunity compared to when no information is available about the likelihood of an antagonistic response. It follows then that OT should inhibit behavioral inhibition imposed by the threat of an agonistic response, thereby indirectly facilitating social approach behavior or any other individually preferred social behaviors typically inhibited by the possibility of antagonism. Importantly, the effects of honest affiliative signals on a receiver’s estimate of agonistic interaction may vary as a function of the prior probability of such interactions. In social systems in which agonism is generally low, as in more egalitarian social systems, OT may have little effect compared to more hierarchical social systems.

## The adaptive component process model

**Figure 4:**
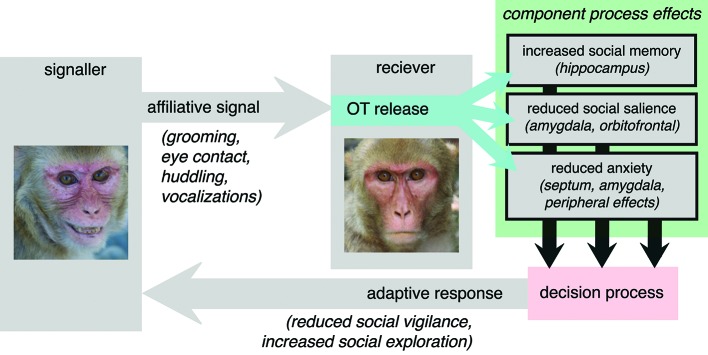
**The adaptive component process model of OT function.** Affiliative signals, particularly those from familiar or socially proximate others, provoke OT release in the receiver. OT then affects behavior via interacting and sometimes complimentary effects on “component processes”. Here, putative component processes are suggested on the basis of OT receptor distribution in the rhesus macaque (Boccia et al., 2007). OT provokes changes at the level of each component processes then indirectly influence signals in decision circuitry responsible for selecting between actions. Ultimately, the response that is generated is an adaptive response to the receipt of honest affiliative signals, informed by the component processes that are regulated by OT.

Given the evolutionarily conserved causes of OT release, we hypothesize that a central function of OT is to act as mediator between affiliative signals in the social environment and the generation of adaptive behavioral responses. This hypothesis places the function of OT squarely within the context of the dyadic interactions that regulate its endogenous release in primates (Figure 4). We argue that OT should function not to release an inevitably prosocial response to an affiliative signal, but rather to release an adaptive and species-typical response, which depends on each species’ unique social and selective pressures. Affiliative signals may recruit different component processes in different species. The vole is an illustrative example. OT is known to be a major determinant of pair bonding in the vole, but it is the distribution of receptors, and thereby recruitment of component processes, that differentiates between monogamous and polygynous voles (Insel and Shapiro, 1992). Specifically, monogamous voles have a comparatively greater density of OT receptors in the nucleus accumbens and prelimbic cortex. Affiliative signals may more effectively elicit reward processes, for example, in monogamous prairie voles, compared to polygynous montane voles, thereby promoting different behavioral responses to identical signals from the two species.

Thus far, we have argued that OT is involved in two behaviors that are both adaptive and released in response to social interactions: it magnifies in/out group decision biases and decreases vigilance for conspecifics. We will now draw two additional parallels between the OT literature and the ethology literature, in which adaptive responses to affiliative signals have been well characterized. First, we review evidence that OT mediates the relationship between affiliative social interactions and the stress-protective endocrine changes that follow those interactions. Second, we predict that OT may mediate the classic effect of group size on vigilance for predators.

### Oxytocin (OT) mediates the neuroendocrine consequences of social interactions

Social interactions are associated with a variety of physiological changes in both humans and other animals. Affiliative interactions, such as grooming and playing with others, are associated with reduced stress responses in baboons, for example (Virgin and Sapolsky, 1997). Moreover, simple receipt of affiliative vocalizations predicts reduced glucocorticoid levels in baboons (Crockford et al., 2008), leading researchers to speculate that the informative content of these honest affiliative signals is protective against stress. In the rhesus macaque, sitting in proximity to others predicts reduced biomarkers of stress (Gust et al., 1993). Receipt of grooming reduces heart rate in monkeys (Boccia et al., 1989; Aureli et al., 1999) and is associated with reduced cortisol levels (Gust et al., 1993). Moreover, grooming others also reduces cortisol metabolite levels in one macaque species (Shutt et al., 2007). Pair bonds in particular may enhance the protective function of these signals. Marmosets, when separated from their mate, exhibit increased urinary cortisol responses to novel stressors. However, when subjected to the same stressor in the presence of their partner, no increase in cortisol levels is observed (Smith et al., 2010). Among the various neural and endocrine changes that occur during affiliative social interactions, the most specific of these is arguably OT.

Several detailed reviews have argued that OT mediates the relationship between social interactions and their physiological benefits (Uvnäs-Moberg, 1998; Insel and Young, 2001). Exogenous OT delivery is sufficient to produce many of the same endocrine and arousal effects as affiliative social interactions. In rats, centrally-delivered OT blunts hypothalamic-pituitary-adrenal (HPA) axis activity, as indexed by reduced corticosterone levels in response to noise stress (Windle et al., 1997). Similarly, in squirrel monkeys chronic OT treatment blunts the typical increase in HPA axis tone in response to social isolation (Parker et al., 2005). In rats, OT provokes prolonged reductions in blood pressure (Petersson et al., 1996) and upregulates the activity of the inhibitory noradrenaline autoreceptor (Petersson et al., 1998). In humans, higher OT levels are correlated with reduced heart rate and blood pressure (Light et al., 2005). Finally, there is direct functional evidence for the hypothesis that OT informs the relationship between social interactions and reduced stress in humans. OT modulates the relationship between affiliative social interactions and reduced cortisol levels (Heinrichs et al., 2003). Future work will be necessary to determine whether OT plays a modulatory or even mediating role in these effects in non-human models of social support, in which invasive and transient blockades of OT signaling can be employed to determine whether OT is necessary for the protective health benefits of social interactions.

### Oxytocin (OT) may mediate the effect of social groups on vigilance

Intriguingly, some of the same social behaviors that predict reduced stress also predict other behaviors, such as reduced vigilance for predators. Therefore, we now examine evidence that OT may be involved in regulating predator vigilance, elucidating one testable prediction that naturally springs from the hypothesis that the fundamental role of OT is to mediate the relationship between affiliative interactions and their behavioral consequences.

In ethology, “vigilance” is a state of scanning the environment for threat, be it a predator or a threatening social partner (Pulliam, 1973; Roberts, 1996). However, vigilance is opportunistically costly. Attention can, by definition, only be directed towards one target at a time, and time spent scanning the environment for threat is time not spent foraging for food, grooming, or pursuing other goals. It is maladaptive to maintain a state of high vigilance when it is not warranted. Indeed, many species reduce their levels of vigilance when predation threat is low (Hunter and Skinner, 1998). Vigilance also decreases as social group size increases (Roberts, 1996). Traditionally, this is interpreted as a “many eyes” effect in which vigilance is reduced because more individuals can detect and orient the group to sources of threat as group size increases (Pulliam, 1973). However, this hypothesis does not address the neurobiological mechanisms that mediate the effects of social grouping on vigilance.

Larger groups provide not only more eyes, but also more opportunities for affiliative social interactions (Dunbar, 2010). Moreover, social interactions may themselves predict variation in vigilance. Neighbor proximity is a better predictor of vigilance than group size in many species (Roberts, 1996) and controlling for neighbor distance may reduce or eliminate the effect of group size on vigilance (Pöysä, 1994). Similarly, vigilance is low during grooming in monkeys (Maestripieri, 1993; Cords, 1995). Unfortunately, rates of vigilance have only been studied in the grooming monkey, not the recipient of grooming, so it remains possible that this effect is due solely to attentional competition and not to the hormonal changes that accompany grooming (Maestripieri, 1993). Future research is needed to determine whether rates of vigilance are similarly reduced in the animal being groomed, who experiences little attentional competition but significant neuroendocrine changes (such an effect would come as no surprise to anyone who has observed a bout of grooming). It will also be critical to examine the time course of these effects in order to determine whether grooming suppresses vigilance through direct attentional competition, which would be an instantaneous effect on vigilance, or though neuroendocrine changes, which would continue to shape behavior long after the grooming interaction ends.

It is not simply the proximity or interaction with a neighbor, however, that determines the effect of group size on vigilance. Rather, the effect of others on vigilance is modulated by the relationship between the vigilant individual and the other; for example, the effect of neighbor proximity on vigilance behavior is modulated by partner familiarity (Kutsukake, 2006; Macintosh and Sicotte, 2009). Similarly, OT release can be modulated by the quality of the relationship between signaler and recipient: more OT is released during grooming when partners are frequent groomers, compared to when they are only distantly related (Crockford et al., 2013). These striking parallels between known determinants of vigilance behavior and the effects of OT provide strong justification for evaluating the role of this peptide in vigilance.

There is also some limited empirical evidence in support of this link. First, OT reduces vigilance behaviors, defined as instances of rearing up onto hindquarters, during noise stress in the rat (Windle et al., 1997). Second, in the rhesus macaque, social vigilance decreases following OT delivery (Ebitz et al., 2013; Parr et al., 2013). However, it remains unknown whether OT reduces vigilance for predatory threat in primates, though the anxiolytic effects of the peptide and its effects on the amygdala are consistent with this idea. Thus, future work is necessary to determine whether OT suppresses predator vigilance and, if so, whether it mediates the relationship between social grouping and vigilance in nature.

## Concluding remarks

The complex effects of OT on social decision-making highlight the need to dissociate the cause from the consequence of OT release. While OT appears to be consistently released in response to positive social interactions, it does not have consistently prosocial consequences. Rather, OT appears to make decisions more prosocial only in circumstances that already evoke prosocial behavior. Instead of a hardwired means by which prosociality begets prosociality, OT provides an internal signal that reflects affiliative interactions and which can, in turn, shape social behavior in sometimes counterintuitive but potentially adaptive ways.

The interactionist component process model provides an important and insightful framework for probing the behavioral effects of OT, but additional work is needed to identify the component processes within each species. Future updates to this model should incorporate what is known about OT receptor distribution and local neural function. In particular, examining the behavioral effects of local OT delivery will prove insightful in updating this model. By injecting OT directly into the cortical and subcortical structures in which its receptors are found, the component effects of OT can be identified and dissociated in terms of their effects on behavior. Research utilizing this technique has shown great promise in rodents (Huber et al., 2005; Guzmán et al., 2013). Continuing this line of inquiry in primates will provide insight into both the mechanism of OT’s behavioral effects, but also into the function of these structures in regulating the comparatively complex social behaviors and decisions of primates.

Additionally, comparisons between the behavioral effects of OT and other agents with known mechanistic consequences will have profound consequences for our understanding of OT function. For example, a substantial open question in the literature is whether the behavioral effects of OT require a central mechanism, or, rather, if any are due to changes in peripheral arousal (Kemp and Guastella, 2011). It is possible that some of the apparently complex cognitive effects of OT, such as changes in social decisions or attention, may be more simply due to changes in arousal or task engagement. Comparing the social attentive effects of OT and beta-blockers, for example, might provide insight into this question, as will careful analyses of the level of task engagement before and after OT.

Response time is too infrequently reported in studies of the decision-making effects of OT, largely due to experimental designs not optimized for the collection of this information. However, this data is essential for understanding decision-making effects and will prove critical in the development of computational models of OT’s effects on social decision-making. Future studies should consider collecting and reporting this data. The few studies that have reported response times indicate that OT slows response times when subjects are making reward-sharing decisions (Chang et al., 2012), classifying emotional stimuli (Petrovic et al., 2008; Di Simplicio et al., 2009), or making decisions to seek social information (Ebitz et al., 2013). However, it does not seem that OT has uniformly sedative effects on response time. For example OT speeds response time when attending to social stimuli would slow performance instead of facilitating it (Ebitz et al., 2013).

The OT literature is truly unique in both the breadth of species studied and the diversity of methodological traditions that have conducted work in this domain. Though we have attempted to draw parallels across this vast literature, and others have previously made admirable contributions to understanding the role of OT across species (Insel and Young, 2001; Donaldson and Young, 2008), future work on OT is poised to make truly interdisciplinary progress in understanding the function of this peptide. In particular, direct comparisons of behavioral effects across phylogenetically distant species may prove informative, as well as collaborations between groups hailing from distinct research traditions. Understanding OT will further not only our knowledge of this fascinating peptide, but also our understanding of the sociality that unites so many species.

## Author contributions

R. Becket Ebitz formulated the hypotheses with input and guidance from Michael L. Platt. R. Becket Ebitz and Michael L. Platt wrote the manuscript.

## Conflict of interest statement

The authors declare that the research was conducted in the absence of any commercial or financial relationships that could be construed as a potential conflict of interest.
